# Preliminary Survey of *Alternaria* Toxins Reduction during Fermentation of Whole Wheat Dough

**DOI:** 10.3390/microorganisms8020303

**Published:** 2020-02-21

**Authors:** Elizabet Janić Hajnal, Lato Pezo, Dejan Orčić, Ljubiša Šarić, Dragana Plavšić, Jovana Kos, Jasna Mastilović

**Affiliations:** 1 Research Center for Technology of Plant Based Food Products, Institute of Food Technology, University of Novi Sad, 21000 Novi Sad, Serbia; ljubisa.saric@fins.uns.ac.rs (L.Š.); dragana.plavsic@fins.uns.ac.rs (D.P.); jovana.kos@fins.uns.ac.rs (J.K.); jasna.mastilovic@fins.uns.ac.rs (J.M.); 2 Institute of General and Physical Chemistry, University of Belgrade, 11000 Belgrade, Serbia; latopezo@gmail.com; 3 Department of Chemistry, Biochemistry and Environmental Protection, Faculty of Sciences, University of Novi Sad, 21000 Novi Sad, Serbia; dejan.orcic@dh.uns.ac.rs

**Keywords:** alternariol, alternariol monomethyl ether, tenuazonic acid, fermentation, sourdough, reduction, LC-MS/MS, mathematical modelling

## Abstract

The aim of this study was to investigate the fate of the most common *Alternaria* toxins found in wheat—tenuazonic acid (TeA), alternariol (AOH), and alternariol monomethyl ether (AME) during sourdough processing. For this purpose, spiked whole wheat flour, 3% sourdough starter, 0.5% of baker’s yeast, and 105% of water calculated on flour weight as a base were used as raw materials. Spiked whole wheat dough was fermented for 4 h, 8 h, 12 h, 24 h, and 48 h at 25 °C, and at each point the fermented dough samples were taken, frozen, lyophilized, grounded, and stored until further analysis. To study the effect of sourdough processing on TeA, AOH and AME content, the validated method of high-performance liquid chromatography coupled to tandem mass spectrometry (LC-MS/MS) for these mycotoxins was used. Mathematical models of *Alternaria* toxins reduction were developed in the form of Four Parameter Logistic Regression function. The maximum reduction of TeA, AOH, and AME levels was archived at 48 h of dough fermentation at 25 °C compared with dough after kneading (0 h). Under these conditions, a reduction of the toxin levels of 60.3 %, 41.5%, and 24.1% was observed for TeA, AOH, and AME, respectively.

## 1. Introduction

During cultivation of cereal crops, cereals are exposed to different weather conditions, which may affect the proliferation of toxigenic filamentous fungi and infection of cereal crops by fungi belonging mainly to the genera *Aspergillus*, *Penicillium*, *Fusarium,* and *Alternaria* [1]. These fungi are capable to produce a large number of different secondary metabolites—mycotoxins, which represent the most commonly occurring chemical contaminants of the cereal crops. Fungi of the genus *Alternaria* are considered as a group of ubiquitous plant pathogens, which can infect crops in the field, and saprophytic species, which can cause post-harvest decay of plant products [2,3], due to their tolerance to low temperature and water stress conditions, i.e., due to their environmental adaptability. *Alternaria* species produce more than 70 different mycotoxins, but the most relevant are tenuazonic acid (TeA), alternariol (AOH), alternariol monomethyl ether (AME), tentoxin (TEN), and altenuene (ALT) [4,5]. Due to their frequent occurrence in food, and their possible harmful effect (genotoxic potential) on human and animal health [4,6], they became compounds of possible concern. However, *Alternaria* toxins in food and feed are not yet regulated, mainly as a consequence of the shortage of toxicological and exposure data, so the limited available data do not allow a comprehensive risk assessment with an acceptable degree of certainty.

The major share of produced wheat (*Tritricum aestivum*) as the second most produced cereal worldwide [7,8] is subjected to the milling process that converts it to flour, which is processed into various foods such as breads, pasta, noodles, and cakes. These wheat-based products represent a significant share of the human diet [9]. Production of bread and some bakery products, include the fermentation step to ensure the desired quality of bakery products. Based on the research literature [10,11] during both yeast fermentation (*Saccharomyces cerevisiae*) and sourdough fermentation (lactic acid bacteria), acidification occurs, as well as enzymatic action. According to Banu et al. [12] and Serrano et al. [13] acid conditions might also have an impact on mycotoxin binding, transformation, and/or degradation processes. Based on so far published studies reviewed by Schaarschmidt and Fauhl-Hassek [14] regarding the fate of mycotoxins during the fermentation step in the baked goods production, the yeast-based process was found in the literature to be the most frequently studied, followed by some studies which examined the effect of fermentation using lactobacilli (solely or in combination with yeast) on mycotoxins. However, the knowledge of the fate of mycotoxins during wheat dough fermentation is largely limited to *Fusarium* toxins, in particular, to deoxynivalenol [14]. Generally, the data found in the literature on the effect of fermentation on mycotoxin levels are contradictory, ranging from decrease to no effect to even increase of examined mycotoxins compared to their initial concentration in the raw materials.

However, so far, no data is available regarding the fate of *Alternaria* toxins during fermentation of wheat dough. Thus, the aim of this study was to investigate the fate of the most common *Alternaria* toxins found in wheat—TeA, AOH, and AME during sourdough processing.

## 2. Materials and Methods

### 2.1. Materials

The whole wheat flour sample was prepared by milling of the entire kernel of wheat with a pilot scale stone mill. The batch of whole wheat flour was well mixed in a twin shaft paddle mixer (Model SLHSJ0.2A, Muyang, China) before taking samples for analysis and experiment. Mixing homogeneity of whole wheat flour was assured by Microtracer method (RF-blue group) described in detail by Pezo et al. [15], using external tracers for mixing homogeneity testing, and quantitatively expressed by relative standard deviation value. In brief, Microtracers (Micro Tracers, Inc., San Francisco, USA) are small coloured ferromagnetic particles with exactly defined size (297 mm) and number of particles per gram. Microtracer was added in whole wheat flour in a ratio of 1:10,000. After mixing and sampling, Microtracer particles were extracted from the samples by a magnetic separator (Rotating detector model, Microtracer Inc., San Francisco, USA) and the separated Microtracer particles was dissolved with 7% Na_2_CO_3_ solution. The colour intensity (reflecting the concentration of Microtracer particles) has been determined by spectrophotometer analysis, at a wavelength of 630 nm (Janway Ltd., Stone, Staffordshire, UK). Also, eight subsamples were taken for analysis of investigated *Alternaria* toxins levels. The whole wheat flour sample was analyzed before the spiking procedure, in order to confirm that it is a blank sample without any of the examined *Alternaria* toxins.

### 2.2. Fermentation Procedure

For liquid sourdough preparation 300 g of spiked whole wheat flour (100 μg kg^−1^ of each TeA, AOH, and AME in flour), 3% of sourdough starter (Ernst Böcker GmbH, Minden, Germany), 0.5% of baker’s yeast, and 105% of water calculated on flour weight as a base were used as raw materials. All ingredients were mixed using mixer (DIOSNA, Dierks & Söhne, GmbH, Germany) at low speed (85 rpm min^−1^) for 5 min. Spiked whole wheat dough was fermented for 4, 8, 12, 24, and 48 h at 25 °C, and at each point, the fermented dough samples were taken, frozen and lyophilized for 72 h (Martin Christ GmbH, Osterode am Harz, Germany) at a temperature gradient from −30 °C to +30 °C. The process was controlled in such a way that when pressure in the system reached its minimum value (0.05 mbar), temperature was increased by 5 °C. When the temperature reached +30 °C, the fermented dough samples were left at that temperature until the end of process (72 h). The lyophilized whole wheat dough samples were ground using a laboratory mill (KnifetecTM 1095 mill, Foss, Hoganas, Sweden), and stored until further analysis of the examined *Alternaria* toxins. Further, at each point, the fermented dough samples were taken for determination of the total number of lactic acid bacteria and the total number of yeasts. Also, at each point, the pH of fermented dough was measured using pH-food control meter with penetration electrode, model HI 99161 (HANNA Instruments, Limena, Italy). The above mention procedures were also applied to the whole wheat dough immediately after kneading (0 h).

### 2.3. Sample Preparation

The modified method by Siegel et al. [16], described in detail in our previous studies [17], was used for sample preparation. In brief, approximately 1 g (exact weights known) of homogenized sample was mixed with 7 mL of water. Afterwards, 2 mL of 2 mol/L aq. HCl and 5 mL of ethyl acetate (EtOAc) were added and shaken (Orbital Shaker PSU-10i, BOECO, Hamburg, Germany) for 45 min, ultrasonicated for 10 min (ATM40-3LCD, Madrid, Spain) and shaken again for 45 min. The extract was transferred into plastic cuvettes and centrifuged (Centrifuge 5804 R, Eppendorf, Germany) at 5000 rpm for 15 min to achieve complete phase separation. Thereafter, 2 mL of the upper (EtOAc) layer was transferred into glass cuvette and evaporated under a stream of nitrogen (Reacti-Therm I#18821, Thermo Scientific, Bellefonte, PA, USA). The dry residue was dissolved in 1 mL of LC-MS-grade methanol (MeOH) and transferred to an HPLC vial through an Econofilter PTFE (13 mm, 0.2 m) syringe filter (Agilent Technologies, Beijing, China) and stored at −20 °C until analysis.

### 2.4. Moisture Content

Moisture content in wheat samples and in all lyophilized whole wheat dough samples was determined according to ISO 712 [18] and was expressed on the dry basis.

### 2.5. Determination of Lactic Acid Bacteria Count

The lactic acid bacteria count (LAB) was determined according to ISO 15214 [19].

### 2.6. Determination of Total Yeasts and Molds Count

Total count of yeasts and molds (TYC) was enumerated according to ISO 21527-1 [20] using the DRBC agar (Himedia, India).

### 2.7. LC-MS/MS Analysis

*Alternaria* toxins (TeA, AOH and AME,) were quantified by high performance liquid chromatography coupled to tandem mass spectrometry (LC-MS/MS) using our previously published method without any modifications, including the equipment, chemicals, and materials [17].

### 2.8. Method Validation

Method validation was performed in terms of matrix effects, linearity, trueness, precision, the limit of detection (LOD) and the limit of quantification (LOQ), by the same procedure, as were described in detail in our previous study [17,21] by an in-house quality control procedure following the guidelines of Commission Decision EC 657 [22].

### 2.9. Alternaria Toxins Determination

*Alternaria* toxins were quantified by external matrix-matched calibration procedure (separate calibrations were prepared for both whole wheat flour and fermented dough samples) in order to eliminate the effect of the matrix. Matrix-matched calibration curves were constructed in the concentration range from LOD to 100 µg kg^−1^ for TeA, AOH and AME, respectively. The calculated concentration of *Alternaria* toxins were corrected for sample preparation recovery (*R*_E_), and were expressed on a dry weight basis. All samples were prepared and analyzed in triplicates. The reduction of TeA, AOH and AME were calculated as:

(1) $$\mathrm{Reduction}\;\mathrm{of}\;Alternaria\;\mathrm{toxin}\left(\%\right)=100-\left(C_{x}\times \frac{100}{C_{0}}\right)$$

where *C_x_* is the concentration of *Alternaria* toxins (TeA, AOH and AME) in the fermented dough samples after 4, 8, 12, 24, and 48 h fermentation; *C*_0_ is the initial concentration of *Alternaria* toxins (TeA, AOH and AME) in the spiked dough sample after kneading (0 h).

### 2.10. Mathematical Modelling and Statistical Analysis

The collected data were presented using descriptive statistics tables. The analysis and mathematical modelling was performed using STATISTICA 13.3 [23].

Various mathematical models in sigmoidal form differentiate from each other in the number of parameters used in the observed equation. The most common forms of the three-parameter models are the growth functions, like: logistic, Gompertz and Von Bertalanffy, while the most known four-parameter models are: Richards, Weibull and Morgan–Mercer–Flodin [24,25]. The Gompertz function is a nonlinear, sigmoidal-growth function, which was developed by Gompertz [26], and it was used for the evaluation of mortality rates of microorganisms. It assumes the form:

(2) w(t)=a⋅exp(−exp(b−c⋅t))

where: *w*(*t*) is the sigmoidal-growth function, a=w(∞), i.e., the value of w where *t* approaches infinity, while b is accounted for the for the slope of the curve, and c is a parameter which describes the point of inflection of the curve.

Several sigmoidal models proposed by Romano [27] and Romano et al. [28] derive from the mathematical model which was originally developed for describing the bacterial growth in pH-controlled batch cultures [29]. These models presented a comparison of sigmoidal models and also demonstrated that the Gompertz model was the best compromise between quality of fit and model complexity. These studies also show the possibility of the modified Gompertz model to be parameterized related to the leavening process. According to the model each fermentation curve exhibits three steps: the induction, the growth and the stationary growth.

In this study, the reduction of three *Alternaria* toxins (TeA, AOH and AME), pH value, and total LAB and total yeast count during the fermentation of whole wheat flour was investigated, according to the four-parameter sigmoidal mathematical model (also known as the Four Parameter Logistic Regression function) written in the form:

(3) $$y(t)=d+\frac{a-d}{1+{\left(\frac{t}{c}\right)}^{b}}$$

where *y*(*t*) presents the reduction of TeA, AOH or AME toxins during the process, as well as the predicted pH value of dough during the process, the total LAB count or the total yeast count.

The four-parameter logistic regression function is suitable for biologic systems, which fits data to the S-shaped curves. The regression coefficients which participate in the function could be described as follows: a - the minimum value that was obtained (close to *t* = 0), d - the maximum value that could be obtained (t=∞), c - the inflection point (the point on the S-shaped curve between a and d) and b = the Hill’s slope of the curve (the steepness of the curve at point c).

The sigmoidal mathematical models were fitted to the experimental data, and six models were developed to relate the responses (reduction of TeA, AOH and AME content, pH value, total LAB and total yeast count) and the single process variables (time).

The adequacy of the developed models was tested using coefficient of determination (*r*^2^), reduced chi-square (*χ*^2^), mean bias error (MBE), root mean square error (RMSE), and mean percentage error (MPE). These commonly used parameters can be calculated as follows:

(4) $$\begin{array}{c} \chi^{2}=\frac{\sum_{i=1}^{N}{\left(x_{\exp,i}-x_{pre,i}\right)}^{2}}{N-n},\;RMSE={\left[\frac{1}{N}\cdot \sum_{i=1}^{N}{\left(x_{pre,i}-x_{\exp,i}\right)}^{2}\right]}^{1/2}\\ MBE=\frac{1}{N}\cdot \sum_{i=1}^{N}\left(x_{pre,i}-x_{\exp,i}\right),\;MPE=\frac{100}{N}\cdot \sum_{i=1}^{N}\left(\frac{\left|x_{pre,i}-x_{\exp,i}\right|}{x_{\exp,i}}\right) \end{array}$$

where *x_exp,i_* stands for the experimental values and *x_pre,i_* are the predicted values obtained by calculating from the model for these measurements. *N* and *n* are the numbers of observations and constants, respectively.

## 3. Results and Discussion

### 3.1. Evaluation of the LC-MS/MS Method

To study the effect of sourdough processing on TeA, AOH, and AME content, a validated LC-MS/MS method for these mycotoxins was used. The validation data of the analytical method for the determination of selected *Alternaria* toxins are given in Table 1. During the validation study, matrix-matched calibration (MMC) standards were used to compensate for the matrix effect, i.e., signal suppression or enhancement of the studied *Alternaria* toxins in the whole wheat flour and fermented dough matrices. Slight signal suppression was observed for AME in both matrices, while the matrix effect for AOH was not observed in any of the matrix. Tenuazonic acid showed signal enhancement in whole wheat flour samples, while in the fermented dough matrix, it showed slight signal suppression. 

The method exhibited good linearity, with correlation coefficients (*r*^2^) above 0.9918.

Trueness was evaluated through recovery studies. The overall method recoveries (*R*_A_) and the sample preparation recoveries (*R*_E_) for target analytes were calculated as were described in detail in our previous study [17,21]. It can be seen that the *R*_A_ and the *R*_E_ for all target analytes were above 70% in both matrices. 

Precision for whole wheat flour and fermented dough, expressed as the repeatability and within-laboratory reproducibility (Table 2), gave RSD values within the range of 4.0–11.3% and 5.7–16.3%, respectively, fulfilling the criteria of RSD ≤ 20% and indicating a good precision of the analytical method.

### 3.2. Reduction of Alternaria Toxins during Fermentation of Whole Wheat Dough

**Figure 1:**
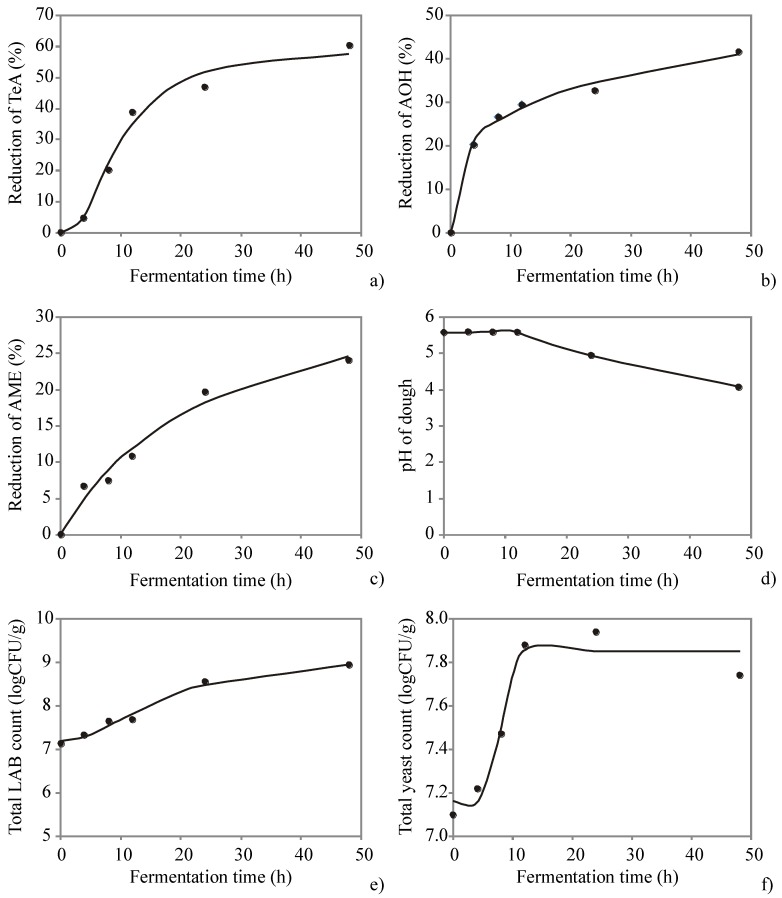
Resulting plots for the model of the leavening process of doughs: (**a**) reduction of tenuazonic acid (TeA), (**b**) reduction of alternariol (AOH), (**c**) reduction of alternariol monomethyl ether (AME), (**d**) pH of the dough during the process, (**e**) total lactic acid bacteria (LAB) count, (**f**) total yeast count.

The capability of the developed models to predict the experimental data during the fermentation is shown in Figure 1. The points plotted in graphics represent the measured values of the observed parameters, while the solid lines connect the points predicted by the mathematical models.

As can be seen in the Figure 1 a–c, the dynamic and the rate of reduction differ among observed *Alternaria* toxins (TeA, AOH, and AME) during prolonged fermentation of whole wheat dough based on the action of both *Saccharomyces cerevisiae* (baker’s yeast) and lactic acid bacteria (sourdough fermentation). In comparison with content of examined *Alternaria* toxins in dough after kneading (0 h), during fermentation of whole wheat dough, the content of TeA was reduced gradually (Figure 1a), and after 48 h of fermentation reduction of 60.3% of this contaminant was achieved. Conversely, the content of AOH was reduced by more than 20% during the first 4 h of fermentation (Figure 1b), but afterward, the reduction of AOH occurred very slowly. At the end of the fermentation of the whole wheat dough (48 h) the observed reduction rate of AOH was 41.5% compared with dough after kneading (0 h). The content of AME was reduced gradually and slowly (Figure 1c), and during 48 h of fermentation of whole wheat dough, a reduction rate of only 24.1% was reached. During fermentation, pH values of whole wheat dough was at the same level up to half of fermentation time, while decrease of pH in the dough was observed at the end of fermentation process (Figure 1d), since total count of LAB increased (Figure 1e) by an order of magnitude, producing organic acids that reduced the pH value of the fermented dough. The total yeast count increased during prolonged fermentation (Figure 1f), while after 24 h of fermentation decrease of total yeast count was observed. Obtained results indicate that the long-lasting sourdough fermentation results in significant decrease of some of the examined *Alternaria* toxin content in dough. However, the obtained results in this study could not be completely compared to the published data, since to the best of authors’ knowledge there is no previously published study regarding the fate of *Alternaria* toxins during long-lasting sourdough fermentation. Certainly, the results obtained in this study confirm the previous findings by other authors (summarized by Schaarschmidt and Fauhl-Hasse [14]) that the changes in mycotoxin concentrations during fermentation processes is largely dependent on several factors: type of mycotoxin, initial concentration of mycotoxins, pH of the dough, the dough composition, the fermentation temperature and duration, as well as the present microbial species and strains. Furthermore, recently published review by Agriopoulou et al. [29] summarized data from representative studies on the occurrence, importance, and mycotoxin control strategies regarding there prevention and detoxification in foods including the possible mechanisms of action of some yeast and LAB strains on some of the mycotoxins during fermentation process. Generally, according to published studies, certain bacteria and yeast have the ability to bind mycotoxins in foods or liquids providing reduction of the mycotoxins content in the final products. 

However, so far, only limited information regarding the fate of *Alternaria* toxins in wheat processing chain have been available. Among the very few reported studies, one of them refers to the investigation of the fate of AOH, AME and TeA during wheat cleaning and milling [30]. Wheat cleaning process reduces the content of AOH, AME and TeA by removing screenings and dust from wheat, since *Alternaria* toxins are concentrated in separated impurities, while wheat milling reduces *Alternaria* toxins content in wheat flours, only due to toxins distribution [30]. Siegel et al. [16] investigated the stability of *Alternaria* toxins (AOH, AME and ALT) during the bread-making process by model experiments. After wet baking conditions (spiked whole wheat flour matrix and water) for 45–60 min at 200 °C, or for 30–45 min at 230 °C (the most realistic conditions), no degradation of examined *Alternaria* toxins was observed [16]. Janić Hajnal et al. [31] investigated the fate of TeA, AOH, and AME during the bread-making process in real conditions on micro-scale. After baking step (for 8 min at 250 °C), the content of TeA and AME in bread was at the same level as in the raw material, while the content of AOH was reduced by 34.8%. Further, during the extrusion process, the highest reduction of AOH (87.9%), AME (94.5%) and TeA (65.6%) in whole wheat flour was achieved when high raw material moisture (*w* = 24 g 100 g^−1^), high feeding rate (*q* = 25 kg h^−1^) and medium screw speed (*v* = 390 rpm) were applied [21]. In a recently published study by Janić Hajnal et al. [32] the effect of atmospheric cold plasma treatments on reduction of *Alternaria* toxins content in wheat flour was investigated. The results obtained in this study indicated that longer exposure time at a shorter distance of the cold plasma source from the sample, provide a greater degree of reduction of AOH (60.6%), AME (73.8%) and TEN (54.5%). Comparison of achieved reduction of *Alternaria* toxins in different steps in wheat processing with achieved reduction during whole wheat sourdough fermentation (reductions of 60.3%, 41.5% and 24.1% for TeA, AOH and AME, respectively) points out, that prolonged dough fermentation is more efficient for *Alternaria* toxins reduction than baking, regardless of applied temperature, but its potential for *Alternaria* toxins reduction in comparison to wheat cleaning, separation of white flours, atmospheric cold plasma treatment or extrusion is lower.

Further, the influence of different time on the observed responses was explained through the regression coefficients. Table 3 summarizes the regression coefficients (a, b, c and d) of the observed sigmoidal mathematical models, which explain the trends (the speed and intensity) of the investigated growth processes. The p-value for each term in a regression model tests the null hypothesis that the coefficient is equal to zero (i.e., it produces no effect to the response variable). If a low p-value is obtained (*p* < 0.05), it indicates that the null hypothesis should be rejected. The regression function of reduction of TeA and AME were found statistically significant at *p* < 0.05 level, while the regression function of reduction of AOH, pH value and total LAB and total yeast count were found statistically significant at *p* < 0.01 level.

The presented four-parameter sigmoidal mathematical models appear to be simple, robust and accurate (all coefficients of determination greater than 0.963, according to Table 4). The quality of the model fit was tested and the residual analysis of the developed model was presented in Table 4. The mathematical models had an insignificant lack of fit tests, which means that all the models represented the data satisfactorily. A high *r*^2^ is indicative that the variation was accounted and that the data fitted satisfactorily to the proposed model.

In brief, this study represents the first report about behavior of *Alternaria* toxins during fermentation of whole wheat dough. Obtained results point out that prolonged sourdough fermentation results in reduction of all observed *Alternaria* toxins: TeA, AOH and AME but the responses differ among observed *Alternaria* toxins with the highest final reduction of TeA, the fastest reduction in initial phases of fermentation for AOH and the slow and steady reduction for AME. Relation of these observations to recorded changes of yeast and LAB count and pH indicate that yeast and LAB metabolic processes, as well as the other biochemical processes might have different roles in individual *Alternaria* toxins reduction. Future research should be related to comprehensive studies of the behavior of *Alternaria* toxins during long-lasting sourdough fermentation by examining the effects of different strains of LAB individually and their combinations, as well as the potential of baker’s yeast sole and in combination with different strains of LAB for reduction of *Alternaria* toxins. Particularly, research should be focused on the mechanisms of action of these microorganisms in terms of the possibility of reducing *Alternaria* toxins in a complex system such as fermented dough, in order to successfully apply this knowledge to produce safe bakery products from the aspect of mycotoxins’ presence. Different biochemical mechanisms involved in degradation of *Alternaria* toxins in the process of sourdough fermentation should also be addressed and investigated. Finally, future investigations have to be conducted related to the nature of degradation products of *Alternaria* toxins and the toxicity of these products formed during and after fermentation.

## Figures and Tables

**Table 1:** Recovery data of the employed analytical method based on solvent (*R*_A_) and matrix-matched (*R*_E_) calibration curves and matrix effect (SSE).

Analytes	Concentration Range (µg kg^−1^) *	Overall Method Recovery *R*_A_ (%) **	Sample Preparation Recovery *R*_E_ (%) ***	Matrix Effect SSE (%) ****	LOD/LOQ (µg kg^−1^)
**Whole wheat flour**					
TeA	2.5–100	87.6	70.0	125.3	2.5/7.5
AOH	2.5–100	70.4	70.4	100.1	0.75/2.5
AME	2.5–100	73.4	76.8	95.6	0.1/0.3
**Fermented dough**					
TeA	2.5–100	83.5	96.0	87.0	3.5/11.6
AOH	2.5–100	87.5	86.9	100.7	0.75/2.5
AME	2.5–100	77.3	86.1	89.8	0.1/0.3

TeA—tenuazonic acid; AOH—alternariol; AME—alternariol monomethyl ether; * Range of concentration of analytes for standard, matrix matched calibration curves and calibration curve of spiked samples (µg kg^−1^). ** *R*_A_—Overall method recovery (%) calculated by slope of spiked sample-prepared curve/slope of solvent calibration curve; *** *R*_E_-Sample preparation recovery (%) calculated by slope of spiked sample-prepared curve/slope of matrix-matched calibration curve; **** SSE-matrix effect (%) calculated by slope of matrix-matched calibration curve/slope of solvent calibration curve; LOD—the limit of detection; LOQ—the limit of quantification.

**Table 2:** Precision parameters of selected *Alternaria* toxins.

TeA			AOH		AME	
Spiking level (µg kg^−1^)	RSD (%) * (*n* = 6)	RSDs (%) ** (*n* = 3 × 6)	RSD (%) * (*n* = 6)	RSDs (%) ** (*n* = 3 × 6)	RSD (%) * (*n* = 6)	RSDs (%) ** (*n* = 3 × 6)
**Whole wheat flour**						
25	10.7	13.6	8.8	9.2	7.5	9.0
50	8.3	9.2	5.4	6.8	5.1	6.9
100	7.8	8.9	5.6	6.8	4.0	5.7
**Fermented dough**						
25	11.3	16.3	6.6	12.2	5.5	10.6
50	10.6	12.7	5.5	9.9	4.4	8.8
100	9.1	10.6	5.4	9.3	4.0	8.2

TeA—tenuazonic acid; AOH—alternariol; AME—alternariol monomethyl ether; * Repeatability expressed as RSD (%)—relative standard deviation of 6 replicates at three concentration levels using the spiked whole wheat flour or fermented dough and the matrix-matched calibration (MMC) curve; ** Within-laboratory reproducibility expressed as RSDs (%)—relative standard deviation of 6 replicates at three concentration levels using the spiked whole wheat flour or fermented dough and the MMC curve, over the course of three days, using the same instrument and by the same operators.

**Table 3:** Regression coefficients in tenuazonic acid (TeA), alternariol (AOH) and alternariol monomethyl ether (AME) reduction models and pH value, total lactic acid bacteria (LAB) and total yeast count prediction models.

Coefficient	TeA *	AOH ^+^	AME *	pH ^+^	LAB ^+^	TYC ^+^
a	−0.981 (0.477)	−0.003 (0.017)	0.366 (0.200)	5.587 ^+^ (0.003)	7.183 ^+^ (0.135)	7.160 ^+^ (0.084)
b	2.234 (0.851)	0.334 (0.031)	1.044 (0.565)	7.213 ^+^ (1.267)	1.859 (0.781)	51.607 (1.261)
c	10.010 * (1.730)	93.552 (43.166)	25.580 (3.272)	25.021 ^+^ (0.231)	18.560 (6.363)	8.033 (0.807)
d	59.228 * (6.513)	151.083 (50.718)	37.123 (2.684)	4.066 ^+^ (0.014)	9.258 ^+^ (0.498)	7.853 ^+^ (0.084)

TeA—tenuazonic acid; AOH—alternariol; AME—alternariol monomethyl ether; LAB—the lactic acid bacteria count; TYC—the total count of yeasts and molds. Each value is the mean of six replicates. Standard deviation values are given in parentheses. ^+^ Significant at *p* < 0.01 level, * Significant at *p* < 0.05.

**Table 4:** The ’goodness of fit’ tests for TeA, AOH and AME reduction models and pH value, total LAB and total yeast count prediction models.

Reduction	*χ* ^2^	RMSE	MBE	MPE	*r* ^2^	Skew	Kurt	Mean	SD	Var.
TeA	21.043	2.648	−0.472	0.000	0.979	−0.070	0.489	−0.567	3.181	10.120
AOH	2.566	0.925	−0.119	0.000	0.993	−0.610	−0.775	−0.143	1.121	1.258
AME	3.997	1.154	0.079	0.000	0.963	0.336	−2.857	0.095	1.410	1.987
pH	0.000	0.003	0.000	0.039	1.000	−1.582	2.699	0.000	0.004	0.000
LAB	0.022	0.085	0.004	0.891	0.963	−0.897	−0.098	0.005	0.104	0.011
TYC	0.008	0.051	0.019	0.518	0.983	−0.600	−0.048	0.023	0.057	0.003

TeA—tenuazonic acid; AOH—alternariol; AME—alternariol monomethyl ether; LAB—the lactic acid bacteria count; TYC—the total count of yeasts and molds; *χ*^2^– reduced chi-square; RMSE—root mean square error; MBE—mean bias error; MPE—mean percentage error; *r*^2^—coefficient of determination; Skew—skeweness; Kurt.—kurtosis; SD—standard deviation; Var.—variance.
